# 141. Effectiveness of a Multipronged Approach to Improve Prophylactic Antibiotic Prescribing in Patients Undergoing Trans-Arterial Chemoembolization

**DOI:** 10.1093/ofid/ofab466.141

**Published:** 2021-12-04

**Authors:** Kai Chee Hung, Nathalie Grace Sy Chua, Winnie Lee, Lay Hoon Andrea Kwa, Shimin Jasmine Chung, Leong Sum

**Affiliations:** Singapore General Hospital, Singapore, Not Applicable, Singapore

## Abstract

**Background:**

In our institution, the significant use of broad-spectrum antibiotics for antibiotic prophylaxis (AP) in trans-arterial chemoembolization (TACE) was operator dependent and not evidence based. Hence, an AP guideline was developed with the Department of Vascular and Interventional Radiology and launched in May 2019, following department roadshows and creation of user-friendly electronic AP order sets. We analyzed the effectiveness and outcomes of our multipronged approach towards improving the standardization of AP prescribing.

**Methods:**

This was a retrospective study of TACE procedures from November 2018 to March 2020, pre and post guideline implementation (Figure 1). Single IV cefazolin 2g dose (or IV clindamycin 600mg in the setting of β-lactam allergy) before TACE in patients with an uncompromised sphincter of Oddi was recommended. Patients with active infections prior to TACE were excluded. AP was deemed inappropriate if it deviated from guidelines (antibiotic choice and/or duration). Primary outcome was AP appropriateness and 30-day TACE related infections.

Figure 1. Timeline of our multipronged approach

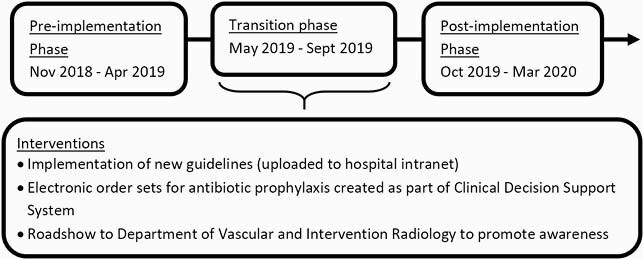

**Results:**

Seventy patients were included. There were no differences in baseline demographics pre and post implementation (Table 1). Following guideline implementation, there was a significant improvement in AP used for TACE. AP appropriateness pre-implementation and post-implementation was 14/31 (45.2%) and 37/39 (94.9%) respectively (*p*< 0.001). Guideline compliant antibiotics were selected more frequently (14 [45.2%] vs 38 [97.4%], *p*< 0.001), and more patients received single dose AP (22 [71.0%] vs 38 [97.4%], *p*=0.004). Of the 18 patients who did not receive guideline recommended AP, 16 (88.9%) received IV ceftriaxone and metronidazole, 1 (5.6%) IV amoxicillin/clavulanic acid, and 1 (5.6%) IV ciprofloxacin. Ten patients received a prolonged course of AP with a median duration of 6 days (IQR 4.3, 6.5). There were no significant differences in 30-day TACE related infections (1 [3.2%] vs 2 [5.1%], *p*=1.000) and 30-day mortality (1 [3.2%] vs 1 [2.6%], *p*=1.000). No patient had surgical site skin infection.

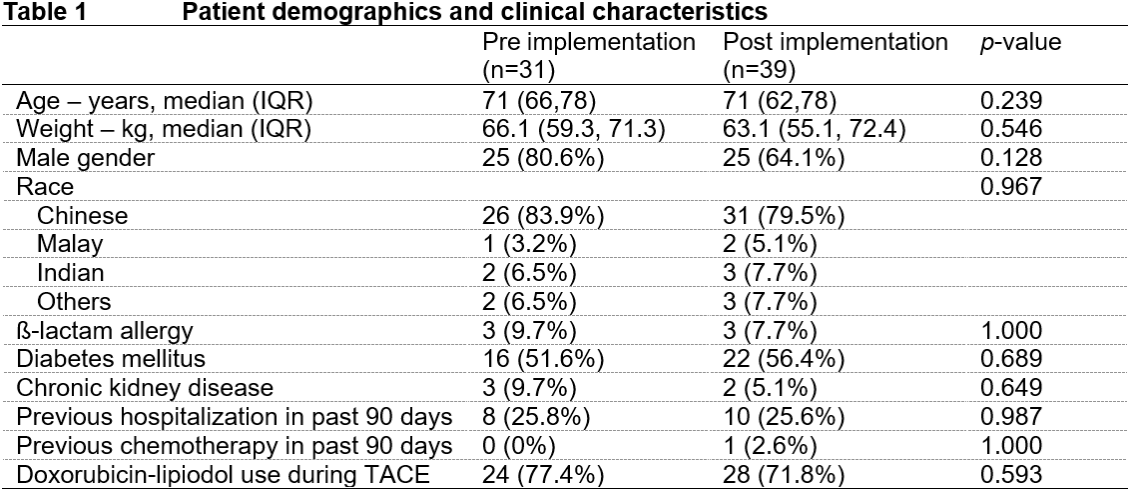

**Conclusion:**

Our multipronged approach improved AP prescribing in patients undergoing TACE. Single dose IV cefazolin prophylaxis for TACE did not compromise safety outcomes in the post implementation review.

**Disclosures:**

**All Authors**: No reported disclosures

